# Integrated Proteomic and Characterization Analyses Revealed the Different Colors of Edible Bird’s Nest

**DOI:** 10.3390/foods15152712

**Published:** 2026-08-01

**Authors:** Si Yang, Bu-Tao Yu, Nan Qian, Guan-Dong Fang, Jing Yao, Dong-Liang Wang, Xiang-Rong Cheng

**Affiliations:** 1 School of Food Science and Technology, Jiangnan University, Wuxi 214122, China; 17746664759@163.com (S.Y.); yubt236@163.com (B.-T.Y.); qiannan202412@163.com (N.Q.); fgd12232023@163.com (G.-D.F.); 2 Jinhua Academy, Zhejiang Chinese Medical University, Jinhua 321000, China; 3 Hebei Edible Bird’s Nest Fresh Stew Technology Innovation Center, Langfang 065700, China; jing.yao@xxdun.com; 4 School of Food Science and Technology, Shihezi University, Shihezi 832003, China

**Keywords:** edible bird’s nest, proteomics, different colors, ICP-MS, Gene Ontology analysis

## Abstract

Edible bird’s nest (EBN) is considered to moisten the lung and nourish yin in China. EBN from different geographical sources vary in morphology, material composition and commercial value. In this study, EBN samples with four different colors were collected, and their protein compositions were analyzed by proteomics technology. Combined with characterization analysis, the physicochemical properties and material composition were analyzed, including physical morphology, protein composition, and metal element composition. Proteomic analysis initially identified 73 differential peptide sequences, of which 59 showed significant abundance differences (*p* < 0.05). After removing redundant peptide assignments, these sequences corresponded to 51 non-redundant proteins exhibiting significant abundance changes among the four EBN color groups. Among them, the beige edible bird’s nest (BEBN) contained a large number of amino acids with iron-chelating ability, which was consistent with its high Fe content. Gene Ontology (GO) analysis revealed that the functions of the differential proteins in EBN were mainly related to glycoprotein hydrolysis. In conclusion, this study elucidated different expressed proteins and their potential functions in EBN with various colors, which provided a basis for production standardization and processing advances in EBN industry.

## 1. Introduction

Edible bird’s nest (EBN) is a nest constructed from the tongue gland secretions of specific swiftlet species, which are primarily sourced from Southeast Asian nations, including Indonesia, Malaysia, Thailand, and Vietnam. EBN has been utilized as a functional food across Asia for over a millennium [1]. EBN is particularly regarded as a traditional medicinal ingredient in China, which is renowned as the ‘caviar of the East’ for its unique flavor and delicate texture [2]. Traditionally served for its moistening the lungs, replenishing yin, and alleviating coughs, its therapeutic effects and medicinal value are documented in over twenty classical Chinese medical texts. Modern pharmacological research has primarily focused on EBN antiviral properties, immune modulation, memory and learning enhancement, improvement of neurodegenerative diseases, and antioxidant effects [3,4].

EBN is rich in multiple nutrients and serves as a primary source of sialic acid, which is often recognized for its high nutritional value in the forms Neu5Ac and Neu5Gc. In recent years, many studies have relied on sialic acid content as the ‘gold standard’ for assessing the quality or grade of EBN [5]. As the sialic acid content cannot be compared visually, the market typically classifies EBN based on size, shape, and color. Consequently, the appearance of EBN directly determines its price. EBNs are categorized by nesting location into cave nests and house nests, by shape into bowl nests, ribbon nests, horn nests, and fragments, or by color into white, yellow, red, or a mixture of red, yellow and white. EBN from natural caves exhibits a varied range of colors, predominantly dark brown or orange-red, whereas artificial production yields only white and yellow varieties [6]. Blood nests (red EBN) command higher market prices than white nests due to perceived superior nutritional value. However, comparative analysis of blood and white nests conducted by Massimo revealed no significant differences in inorganic ash, carbohydrate, or protein content [7]. Furthermore, SDS-PAGE revealed that both blood and white nests possess unique protein fingerprints, indicating that nests from different origins or varieties exhibit distinct protein compositions. However, the formation mechanism of EBN color is still unclear, which may be closely related to the growth environment, the collection method, the inorganic components and environmental factors exposed during the subsequent processing. Nitrite may directly induce color change through nitrosation reaction. Phenolics derived from the environment (bird feces or plant decomposition) are natural pigment precursors that generate quinone pigments through metal ion-catalyzed oxidative polymerization [8], which are potential chemical sources for EBN color. Metal elements (such as Fe and Cu) play a dual role in the formation of color, not only acting as catalysts to accelerate the oxidation of phenolic substances, but also directly coordinating with EBN glycoprotein to form stable colored complexes. Therefore, systematic detection of the distribution of nitrite, phenolics, and metal elements is of key significance for comprehensively analyzing the origin of EBN color.

The primary constituents of EBN (approximately 80–90%) are complex glycoproteins. These glycoproteins comprise a polypeptide backbone with covalently attached oligosaccharide chains, where sialic acid typically resides in a bound form at the terminal ends of these sugar chains. Consequently, variations in the types, quantities, and structural modifications of glycoproteins and their sugar chains across different EBNs collectively generate their unique protein fingerprints, ultimately influencing its quality and functional characteristics. Advances in LC-MS-based proteomics strategies enable protein identification on a large scale and stable discovery of protein biomarkers [9]. The emerging quantitative proteomics technologies allow for systematic investigation of static or non-static states within the proteome-wide expression profile [10]. Proteomic analysis has been employed to characterize signature peptides in various stewed or adulterated EBNs [11,12].

Multiple studies have employed multidimensional analytical techniques such as electrophoresis, spectroscopy, and chromatography to identify protein biomarkers related to the origin, foaming rate, and functional components of EBN [13]. Furthermore, several studies have preliminarily identified potential biomarkers associated with EBN quality, including functional proteins linked to immunomodulation, epidermal growth factor-like activity, and sialic acid metabolism. The different origins, environments, and collection techniques of EBN may have different protein characteristics, reflecting the inherent differences in formation processes of EBN that could ultimately influence nutritional quality and market value. To our knowledge, there is currently a lack of systematic, in-depth research into the relationship between different colored EBNs and their material foundations, such as physical properties, elemental composition, and proteome. Although the four EBNs with various colors originate from the same source, the comparative data through sodium dodecyl sulfate–polyacrylamide gel electrophoresis (SDS-PAGE), liquid chromatography–tandem mass spectrometry (LC-MS/MS), and physicochemical analysis are crucial for understanding the formation of EBN coloration. Consequently, this study selected four different colored EBNs to elucidate differences in indicators such as nitrite content, inorganic metal elements, and protein expression, providing evidence for the relationship between the formation of EBN color and its material basis.

## 2. Materials and Methods

### 2.1. Materials

Bradford Protein concentration assay kit, SDS-PAGE loading buffer, Coomassie Brilliant Blue R-250, and protein molecular weight standard Marker were purchased from Shanghai Biyun Tian Biotechnology Co., Ltd. (Shanghai, China). Formic acid and acetonitrile (Mass spectrometric grade) were purchased from Shanghai Aladdin Biochemical Technology Co., Ltd. (Shanghai, China). Trypsin was purchased from Sigma-Aldrich (St. Louis, MO, USA). Acetone, methanol, sodium carbonate, potassium ferrocyanide and other chemical reagents were purchased from Sinopharm Group Chemical Reagent Co., Ltd. (Shanghai, China).

### 2.2. Samples

The EBN materials comprised white edible bird’s nest (WEBN), creamy white edible bird’s nest (CWEBN), beige edible bird’s nest (BEBN), and pink edible bird’s nest (PEBN). All EBN samples were provided by the Hebei Edible Bird’s Nest Fresh Stew Technology Innovation Center (China). The provider was only responsible for sample supply and did not participate in experimental design, data acquisition, data analysis, interpretation of results, or manuscript preparation. Samples from the four color categories were obtained from the same manufacturer across different production batches. For each category, three independent individual nests were used as biological replicates (*n* = 3). Each individual nest sample was separately de-feathered, ground into a fine powder, and passed through a 60-mesh sieve. The resulting EBN powder was stored in sealed containers at 4 °C until further analysis. Each individual nest was treated as the experimental unit, and technical replicate measurements (where applicable) were averaged before statistical analysis.

### 2.3. Color Difference Determination

The color parameters of each sample were determined using a CR-400 colorimeter (Konica Minolta, Tokyo, Japan). Before measurement, the instrument was calibrated using the standard white calibration plate provided by the manufacturer. Each sample was placed on the standard white plate as the background, and the color values were measured under the CIE Lab* color system. The L*, a*, and b* values were recorded to represent lightness, redness/greenness, and yellowness/blueness, respectively. The total color difference (ΔE) was calculated to evaluate the overall color variation among different samples.

### 2.4. Protein Content Determination

The protein content in each sample was determined using the Coomassie Brilliant Blue method. Each sample powder was dissolved to form a solution of suitable concentration. A 5.0 μL aliquot of the test solution was mixed with 250 μL of Bradford reagent, and the absorbance was detected subsequently at 595 nm after standing for 20 min. Protein content was calculated based on a BSA standard curve [14].

### 2.5. Determination of Total Phenolic Content (TPC)

The TPC in EBN was determined using the Folin–Ciocalteu method. An 80% acetone solution was added at a material–liquid ratio of 1:20, before being ultrasonicated in an ice bath for 5 min and filtered through a 0.22 nm microporous membrane. The operation was repeated twice, and the sample was evaporated until completely dry under reduced pressure at 50 °C using a rotary evaporator. Samples were resuspended in 5 mL of methanol, with 2.5 mL of 10-fold diluted Folin–Ciocalteu reagent. After mixing the samples using a vortex, they reacted in the dark for 5 min. A total of 2 mL of 7.5% Na_2_CO_3_ solution was added afterwards for reaction in the dark for 60 min. Absorbance was measured at 765 nm. A standard curve was prepared using 0.05 mg/mL gallic acid standard solution at concentrations of 0, 0.2, 0.4, 0.6, 0.8, and 1.0 mL. The TPC was calculated and expressed as mg of gallic acid equivalent per gram of EBN (mg/g) [15].

### 2.6. Determination of Nitrite Content

Nitrite content was determined according to a previously reported spectrophotometric method with slight modifications. The sample preparation procedure was performed with reference to GB 5009.33-2016 [16]. A total of 10 g of homogenized EBN sample was added to a 250 mL conical flask. A total of 100 mL of ultrapure water, 5 mL of ammonium buffer solution (pH 9.6–9.7), and 2 g of powdered activated carbon were subsequently added and shaken for 30 min. After transferring to a 250 mL volumetric flask, 2 mL of 150 g/L potassium ferrocyanide solution and 2 mL of 300 g/L zinc sulphate solution were added. The solution was subsequently diluted with water and stood for 5 min. The supernatant was filtered through quantitative filter paper, and the filtrate was reserved. A blank control served as the control group, and the absorbance at 540 nm was measured using a 1 cm quartz cuvette. A 10.0 mL aliquot of the obtained filtrate was transferred to a test tube, followed by the sequential addition of 2 mL of p-aminobenzenesulfonic acid solution and 1 mL of N-(1-naphthyl)ethylenediamine dihydrochloride solution. After mixing thoroughly and standing for 15 min at room temperature for full color development, the absorbance was measured at 540 nm using a 1 cm cuvette against a blank control.

### 2.7. Determination of Protein Secondary Structure

The secondary structure of the test samples was characterized using Attenuated Total Reflection Fourier-transform Infrared (ATR-FTIR) spectroscopy. The scan range was 4000–650 cm^−1^, with 32 scans performed at a resolution of 4 cm^−1^.

### 2.8. Determination of Metal Ions by ICP-MS

A total of 0.2 g of EBN powder was added to a digestion tube with 5 mL of concentrated nitric acid. The heating temperature was set to 120 °C for digestion for 40 min. Then 2 mL of hydrogen peroxide was added before digestion for 30 min until the sample was fully digested. After cooling, the sample was diluted to 50 mL with ultrapure water. A blank sample was prepared using the same digestion method for subsequent testing. Calibration curves were established using multi-element standard solutions before sample analysis, and blank correction was performed to minimize background interference. Each sample was measured in replicate to ensure the reliability and precision of the analytical results. ICP-MS parameters are as follows: high-purity argon as carrier gas, carrier gas flow rate: 1.04 L/min; pump speed: 0.1 r/s; plasma gas flow rate: 15 L/min; auxiliary gas flow rate: 1.2 L/min; RF power: 1600 W; RF voltage: 1.93 V; detection mass numbers: m/z = 7 (Li), m/z = 89 (Y), m/z = 205 (Tl).

### 2.9. Sodium Dodecyl Sulphate–Polyacrylamide Gel Electrophoresis (SDS-PAGE)

The sample was mixed with 2X loading buffer to achieve a final protein concentration of 2 mg/mL. After 5 min boiling, the sample was centrifuged and the supernatant was collected. A total of 10 μL of the sample solution was loaded into the electrophoresis lanes (15% polyacrylamide separation gel and 5% concentration gel). Electrophoresis conditions are as follows: concentration gel voltage: 70 V; separation gel voltage: 140 V. Following electrophoresis, the sample was stained overnight with Coomassie Brilliant Blue R-250. It was decolorized in 7.5% methanol-water-5% glacial acetic acid (4.5:4.5:1) solution until the background was clear. Images were captured using a gel imaging system. Standard molecular weight markers were employed, spanning 10–250 kDa.

### 2.10. LC/MS-MS

For proteomic analysis, proteins were extracted separately from each biological replicate. Briefly, EBN powder was extracted with ultrapure water at a ratio of 1:20 (*w*/*v*) under ultrasonication for 5 min. The protein was sequentially hydrolyzed using Alcalase 2.4 L (6000 U/g, 60 °C, pH 8.5) for 12 h and Papain (10,000 U/g, 50 °C, pH 7.0) for 2 h. Total proteins were precipitated with ice-cold acetone, centrifuged at 15,000× *g* for 45 min, and collected for trypsin digestion. Following enzymatic digestion, peptide samples corresponding to each individual edible bird’s nest were independently subjected to LC-MS/MS analysis. The resulting datasets represented three biological replicates for each color category.

The peptide samples were dissolved in 0.1% formic acid aqueous solution, centrifuged at 12,000 rpm for 10 min, and the supernatant was transferred into sample vials. A total of 500 ng peptide sample was loaded for each LC-MS/MS analysis. Peptide separation was performed using a VANQUISH NEO nano-liquid chromatography system (Thermo Scientific™, Waltham, MA, USA) equipped with a PepMap NEO C18 trap column (5 μm, 300 μm × 5 mm) and an Acclaim PepMap nanoViper C18 analytical column (2 μm, 100 Å, 75 μm × 25 cm). The mobile phase consisted of solvent A (water containing 0.1% formic acid) and solvent B (80% acetonitrile containing 0.1% formic acid). The flow rate was maintained at 0.30 μL/min, and the column temperature was set to 55 °C. The gradient elution program was as follows: 0–1.8 min, 4–4.5% B; 1.8–2.0 min, 4.5–5% B; 2.0–41.0 min, 5–20% B; 41.0–57.0 min, 20–35% B; 57.0–57.5 min, 35–55% B; 57.5–58.0 min, 55–99% B; and 58.0–60.0 min, 99% B.

Following chromatographic separation, peptide ions were introduced into the nano-electrospray ionization (nano-ESI) source and analyzed using an Orbitrap Exploris™ 480 liquid chromatography–mass spectrometry system (Thermo Scientific™). The ion source voltage was set to 2.0 kV, and the capillary temperature was maintained at 320 °C. MS1 spectra were acquired in positive ion mode with a scan range of m/z 350–1500 and a resolution of 60,000. Data were acquired in data-dependent acquisition (DDA) mode, with a cycle time of 2 s between master scans. The MS2 spectra were acquired at a resolution of 15,000, with a fixed first mass of m/z 110. The isolation window was set to 1.6 m/z. Peptide fragmentation was performed using higher-energy collisional dissociation (HCD) at 30% collision energy. The AGC target was set as custom, the maximum injection time was 22 ms, and the dynamic exclusion duration was 45 s.

The mass spectrometer was operated according to the standard calibration procedure provided by the manufacturer. No additional internal calibration standard was introduced during LC-MS/MS acquisition.

### 2.11. Data Analysis

For protein identification, a custom reference database was constructed by filtering and downloading all protein sequences taxonomically assigned to the order Apodiformes (Taxon ID: 8892) from the UniProtKB database (UniProt release 2024_03, containing 23,485 protein sequences). Secondary mass spectrometry data were analyzed using Thermo Proteome Discoverer software (version 3.0) with the following database search parameters: digestion method: trypsin (full); maximum missed cuts: 3; minimum peptide length: 6 amino acid residues; maximum post-translational modifications: 3; primary parent ion mass tolerance: 20 ppm; secondary fragment ion tolerance: 0.02 Da. Peptide post-translational modification settings were as follows: fixed modifications: carbamidomethylation (C). Variable modifications were as follows: methionine oxidation (M), N-terminal acetylation (AnyN-term), methionine loss (ProteinN-termM), methionine loss + acetylation (ProteinN-termM). The FDR (false positive rate) for identified proteins, peptides, and PSM (peptide-sequence matching) was set to 1%.

Protein inference was performed using Proteome Discoverer (version 3.0) based on the principle of parsimony. Peptides were assigned to proteins according to peptide evidence, including unique peptides and razor peptides. Unique peptides exclusively matched to a single protein were used as direct evidence for protein identification, whereas razor peptides shared among multiple protein candidates were assigned to the protein group with the strongest peptide evidence according to the parsimony rule. Proteins sharing indistinguishable peptide evidence were grouped into protein groups to minimize redundant protein assignments. The final protein list was generated after protein grouping and filtered using a protein-level FDR threshold of 1%. For differential analysis, normalized protein abundances were used after removing proteins with missing values across biological replicates. Pairwise comparisons were evaluated using Student’s *t*-test, and *p*-values were adjusted via the Benjamini–Hochberg FDR method. Differentially expressed proteins (DEPs) were filtered using thresholds of adjusted *p*-value < 0.05 and |Log2FC| > 1.00, and visualized using volcano plots.

### 2.12. Statistical Analysis

Experiments were performed in triplicate and data are presented as mean ± standard deviation. Three independent biological replicates (*n* = 3) were prepared for each EBN color category. For physicochemical analyses, repeated measurements obtained from the same biological sample were considered technical replicates, and the average values were used for statistical comparisons. Statistical evaluation and data visualization were performed using GraphPad Prism 8 (GraphPad Software, San Diego, CA, USA) and R software. For proteomic analysis, biological replicates were treated as independent observations for statistical evaluation. One-way analysis of variance (ANOVA) was performed using GraphPad Prism 8 (GraphPad Software, USA) to determine statistical significance. Tukey’s multiple comparison test was subsequently applied as a post hoc analysis to identify significant differences among groups and generate the compact letter displays (a–d), with *p* < 0.05 considered statistically significant. Proteomic data visualization and bioinformatics analyses encompassed principal component analysis (PCA), volcano plot generation, heatmap visualization, and functional enrichment analysis.

## 3. Results

### 3.1. Differences in EBN Appearance

**Figure 1:**
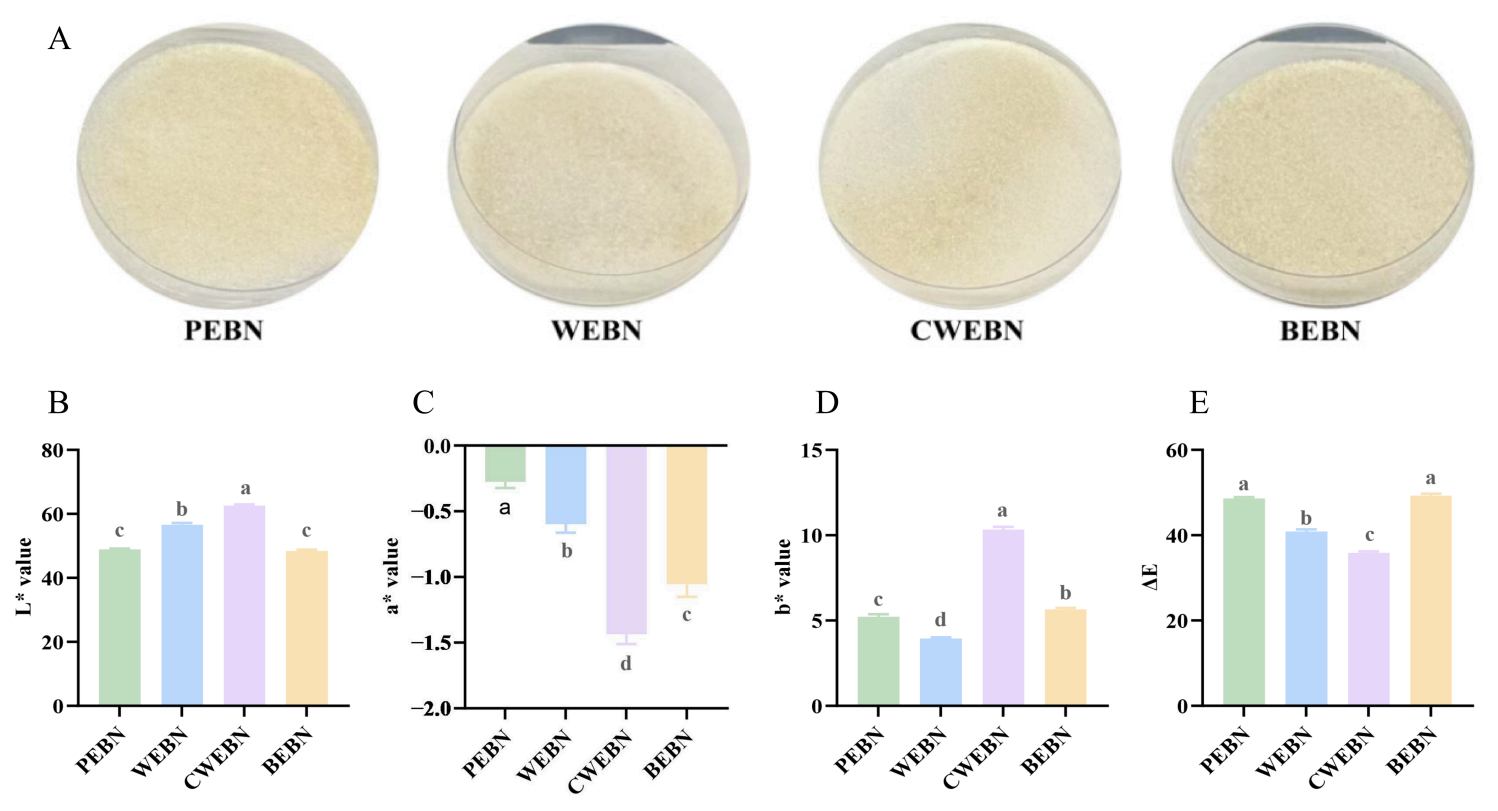
Visual appearance and objective color parameters of the four types of edible bird’s nest (EBN). (**A**) Visual appearance of PEBN, WEBN, CWEBN, and BEBN samples; (**B**) lightness (L* value); (**C**) redness/greenness (a* value); (**D**) yellowness/blueness (b* value); (**E**) total color difference (ΔE). Data are expressed as mean ± standard deviation. Different lowercase letters (a–d) above the bars indicate significant differences among different EBN categories according to one-way analysis of variance (ANOVA) followed by Tukey’s honestly significant difference (HSD) test (*p* < 0.05). L*, a*, and b* values represent the lightness, red-green coordinate, and yellow-blue coordinate, respectively, according to the CIELAB color system.

The appearances of the four types of EBN are shown in Figure 1A. The four types of EBN investigated in this study included PEBN, WEBN, CWEBN, and BEBN. The objective color differences among the four EBN categories were further evaluated based on the ΔE values (Figure 1E). Figure 1B, Figure 1C, and Figure 1D represent the corresponding L*, a*, and b* color parameters, respectively. Significant differences were observed in the ΔE values among the four EBN categories (*p* < 0.05). Pairwise comparisons revealed that the color differences between different EBN groups were statistically significant, indicating that PEBN, WEBN, CWEBN, and BEBN exhibited distinct color characteristics. These results provide quantitative evidence supporting the classification of EBN samples into four color categories.

### 3.2. The Protein Content and Distribution of EBN

**Figure 2:**
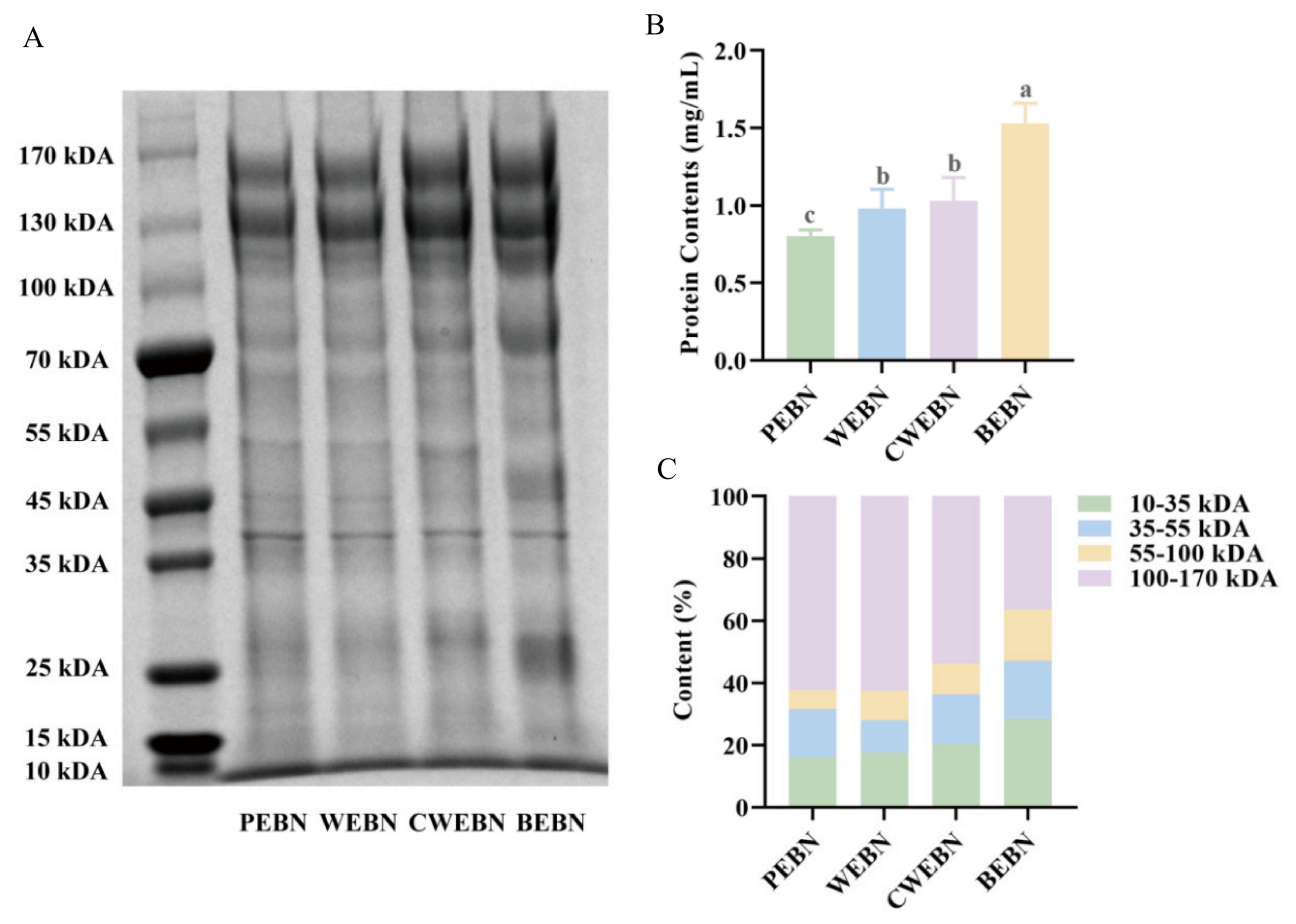
Protein profile, concentration, and molecular weight distribution of the four types of edible bird’s nest (EBN). (**A**) SDS-PAGE analysis of proteins extracted from PEBN, WEBN, CWEBN, and BEBN; (**B**) total protein contents (mg/mL) of the four EBN categories; (**C**) relative percentages of different protein molecular weight fractions (10–35 kDa, 35–55 kDa, 55–100 kDa, and 100–170 kDa). Data in (**B**) are presented as mean ± standard deviation. Different lowercase letters (a–c) above the bars indicate significant differences among different EBN categories according to one-way analysis of variance (ANOVA) followed by Tukey’s honestly significant difference (HSD) test (*p* < 0.05).

To investigate the relationship between the color and protein content of EBNs, this study determined the protein content of PEBN, WEBN, CWEBN, and BEBN (Figure 2A and Figure S1). As shown in Figure 2B, four EBNs showed significant differences in the protein concentration (*p* < 0.05). Among these, the protein concentration of BEBN was higher, and the protein extraction rate was also higher than that of other three EBNs. Furthermore, the molecular weight distribution of proteins in four EBNs was analyzed through SDS-PAGE. The relative molecular weight distribution of proteins in various colored EBNs is shown in Figure 2C. All four types exhibited similar protein band patterns, with a mass range of 10–170 kDa. The distribution was predominantly concentrated between 100 and 170 kDa, with the bands around 130 kDa being particularly distinct. However, a group of common proteins with a range of 35–70 kDa was present. The relative contents of various groups of EBN proteins were normalized. The results are shown in Table 1. Among the three categories (PEBN, WEBN, and CWEBN), proteins within the 100–170 kDa molecular weight range exhibited the highest abundance. In BEBN, proteins in this range constituted 36.41% of the total, followed by the proteins in the 10–35 kDa range at 28.47%. Clearly, BEBN had fewer large-molecule proteins compared to the other three colors, but more proteins in the 10–35 kDa range.

### 3.3. The Nitrite Content of EBN

**Figure 3:**
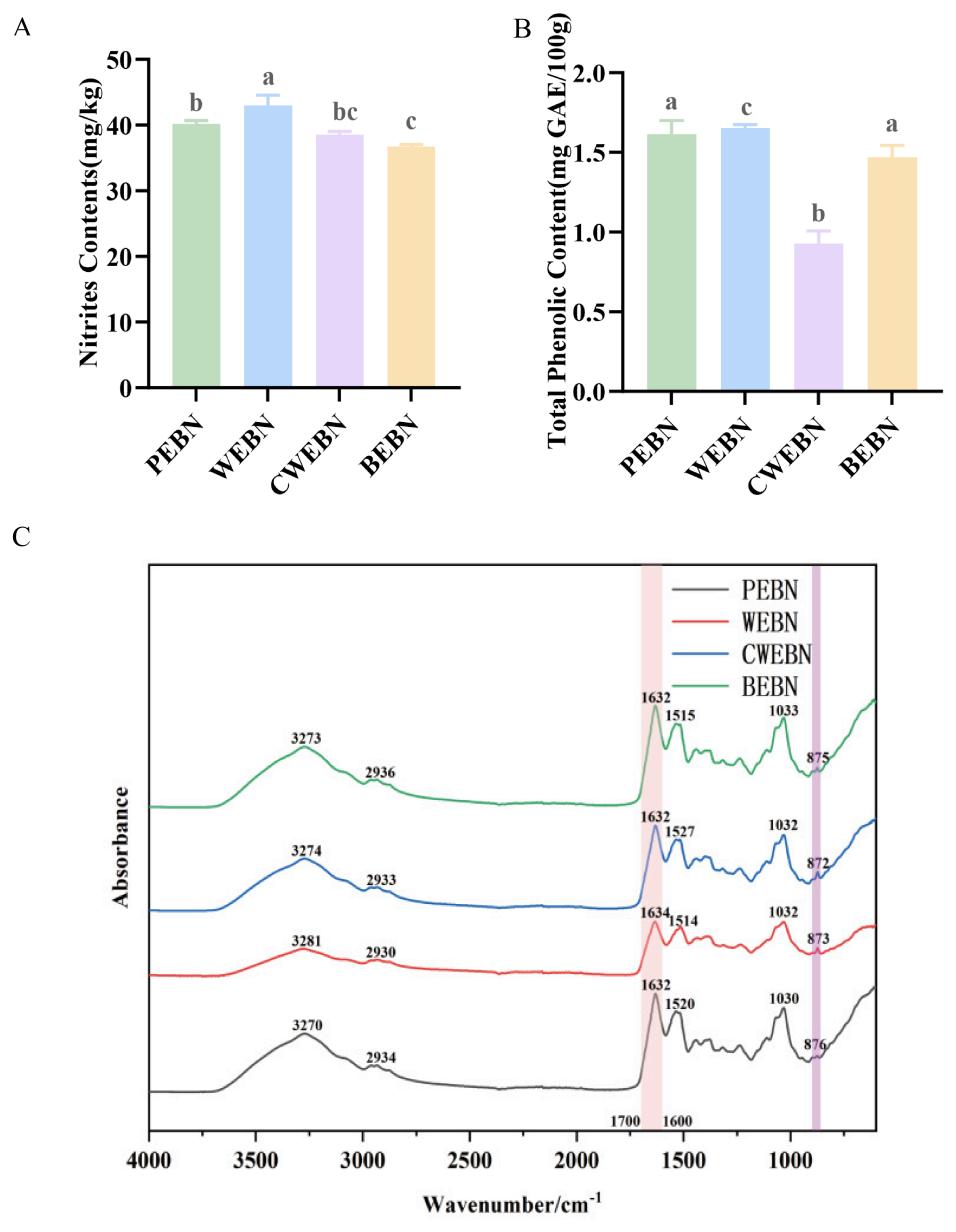
Nitrite contents, total phenolic contents, and FT-IR spectra of the four EBN categories. (**A**) Nitrite contents (mg/kg). (**B**) Total phenolic contents (mg GAE/100 g). (**C**) Stacked FT-IR spectra from 4000 to 500 cm^−1^. Data are presented as mean ± SD (*n* = 3). Different lowercase letters (a–c) above the bars indicate significant differences among different EBN categories according to one-way analysis of variance (ANOVA) followed by Tukey’s honestly significant difference (HSD) test (*p* < 0.05). The shaded regions indicate the characteristic absorption bands of amide I (1632–1634 cm^−1^) and polysaccharide-related vibrations (1030–1033 and 875 cm^−1^), respectively.

The nitrite content of four EBNs was compared. As shown in Figure 3A. Nitrite concentrations in all EBNs ranged from 36.67 to 42.92 mg/kg. As early as August 2011, the Chinese government imposed a ban on imported EBN products from Malaysia due to excessively high nitrite levels detected in these goods. The Malaysian government department has issued a document stipulating that the permissible nitrite content standard is set at 30 ppm, consistent with China’s maximum import and export limits. Nitrite levels in all samples were slightly higher than the limits set by the government due to the natural background levels in raw materials. Among the four samples, WEBN exhibited the highest nitrite concentration at 42.92 mg/kg, while BEBN showed the lowest at 36.67 mg/kg. There were differences in nitrite content among the four types of EBNs.

### 3.4. The Total Phenolic Content of EBN

Figure 3B shows the total phenolic content across different colored EBNs. The overall phenolic content of the four EBNs was relatively low, ranging from 0.93 to 1.65 mg GAE/100 mg. The phenolic content of the WEBN was the highest, followed by the PEBN.

### 3.5. The FT-IR Spectroscopy of EBN

To confirm the distinctions between four different colored EBNs, we compared FT-IR spectra of PEBN, WEBN, CWEBN, and BEBN in the wavenumber range of 4000–650 cm^−1^ (Figure 3C). The EBN spectra exhibited characteristic polysaccharide and polypeptide absorption patterns. The absorption wavelengths of each compound in EBN (1632, 1532, 1033 cm^−1^) were different from the reported values (1655, 1517, 1047 cm^−1^). The absorbance of the EBN samples is 3270–3281 cm^−1^ (peak 1), 2930–2936 cm^−1^ (peak 2), 1632–1634 cm^−1^ (peak 3), 1514–1527 cm^−1^ (peak 4), 1030–1033 cm^−1^ (peak 5), and 872–876 cm^−1^ (peak 6). Among them, the 875 cm^−1^ peak was clearly visible in all four different colors of EBN. Previous studies have shown that this peak is a characteristic peak of true EBN [17]. The peaks from 1 to 6 are common in all FT-IR spectra of EBN, with only minor changes in the wave numbers of some characteristic peaks.

### 3.6. Element Contents of EBN

**Figure 4:**
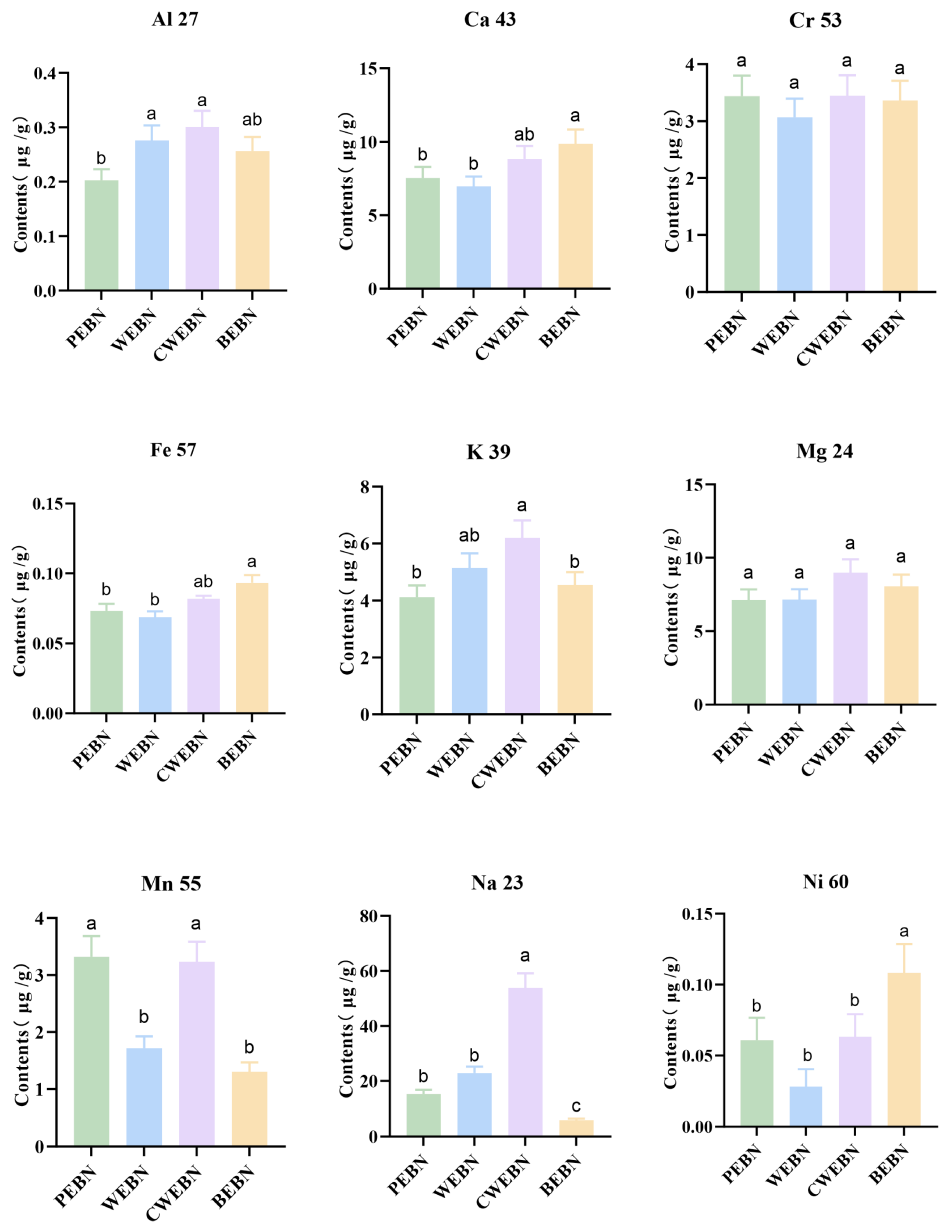
Trace element concentrations of the four EBN categories determined via ICP-MS. Quantitative distribution profiles for individual mineral species, including Al, Ca, Co, Cr, Fe, K, Mg, Mn, Na, and Ni, expressed in standard units of µg/g. Data are presented as mean ± SD (*n* = 3). Different lowercase letters (a–c) above the bars indicate significant differences among different EBN categories according to one-way analysis of variance (ANOVA) followed by Tukey’s honestly significant difference (HSD) test (*p* < 0.05).

The elemental distribution of the four EBN samples is shown in Figure 4. The results are expressed as μg/g of EBN. Na showed the highest content among the detected elements, followed by Mg, K, Ca, and Fe, which is consistent with the findings of Warasri et al. [18]. Significant variations in Na content were observed among different colored EBN samples. Fe content showed significant differences among EBN categories, with BEBN exhibiting relatively higher Fe levels than PEBN and CWEBN. Although Mg and Ni contents were relatively low in the samples, significant differences in Mg content were observed among different colored EBN samples. BEBN showed significant differences in Ni content compared with the other three groups.

**Figure 5:**
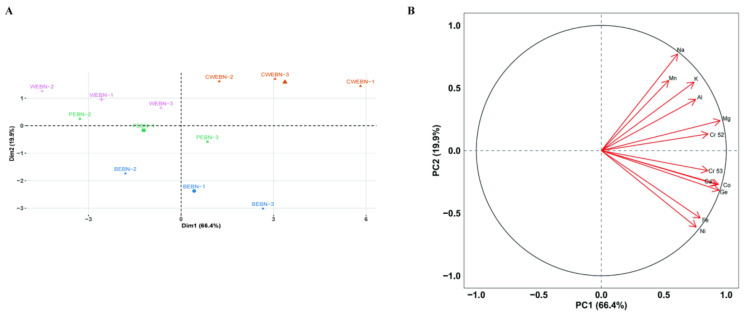
Principal component analysis (PCA) of metal element contents in four types of edible bird’s nest (EBN). (**A**) PCA score plot showing the distribution and clustering patterns of PEBN, WEBN, CWEBN, and BEBN samples based on their metal element profiles. (**B**) PCA loading plot illustrating the contributions of individual metal elements to the principal components. The percentages in parentheses represent the contribution rates of PC1 and PC2 to the total variance.

The principal component analysis (PCA) of standardized mineral element content values in EBN is shown in Figure 5. According to the principle that the cumulative variance contribution rate should reach 80–85% and the eigenvalue should be greater than 1, the number of principal components to be extracted is determined. The eigenvalues of the first three principal components are all greater than 1, at 7.963, 2.386, and 1.317, respectively. The cumulative variance explained reached 97.22%. The eigenvectors estimated from the correlation matrix indicated that PC1 and PC2 accounted for 66.36% and 19.89% of the total variance, respectively. Thus, the first two principal components collectively represented 86.24% of the total variance. PC1 showed significant positive correlations with Ca and Mg. The negative component of PC1 was primarily associated with Ni, Fe, Ge, and Ca, while the remaining variables were linked to the positive component of PC1. PC2 exhibited a significant positive correlation with Na and a positive correlation with K. The negative portion of PC2 was mainly associated with Ni, Fe, and Ge, while the remaining variables correlated with its positive portion. Based on this, the samples were clustered to generate PCA analysis results for different colored EBNs. On the negative portion of PC1 and the positive portion of PC2, PEBN and WEBN cluster closely together. Conversely, CWEBN, situated in the positive regions of both principal components, was not close to the other three colored EBNs. In summary, the mineral composition of CWEBN and BEBN was significantly different. There was no obvious difference between PEBN and WEBN. CWEBN exhibited relatively higher Na, K, and Mg contents, whereas BEBN showed relatively higher Fe and Ni contents.

### 3.7. Proteomics of EBN

A total of 16 LC-MS/MS runs were performed for the proteomic analysis of the different colored EBN samples. The quality and coverage of the proteomic dataset were evaluated based on the available identification parameters, including the numbers of identified protein groups, identified peptides, unique peptides, and sequence coverage. Protein and peptide identification was performed using stringent database search criteria with a false discovery rate (FDR) threshold of 1% at the protein, peptide, and PSM levels.

**Figure 6:**
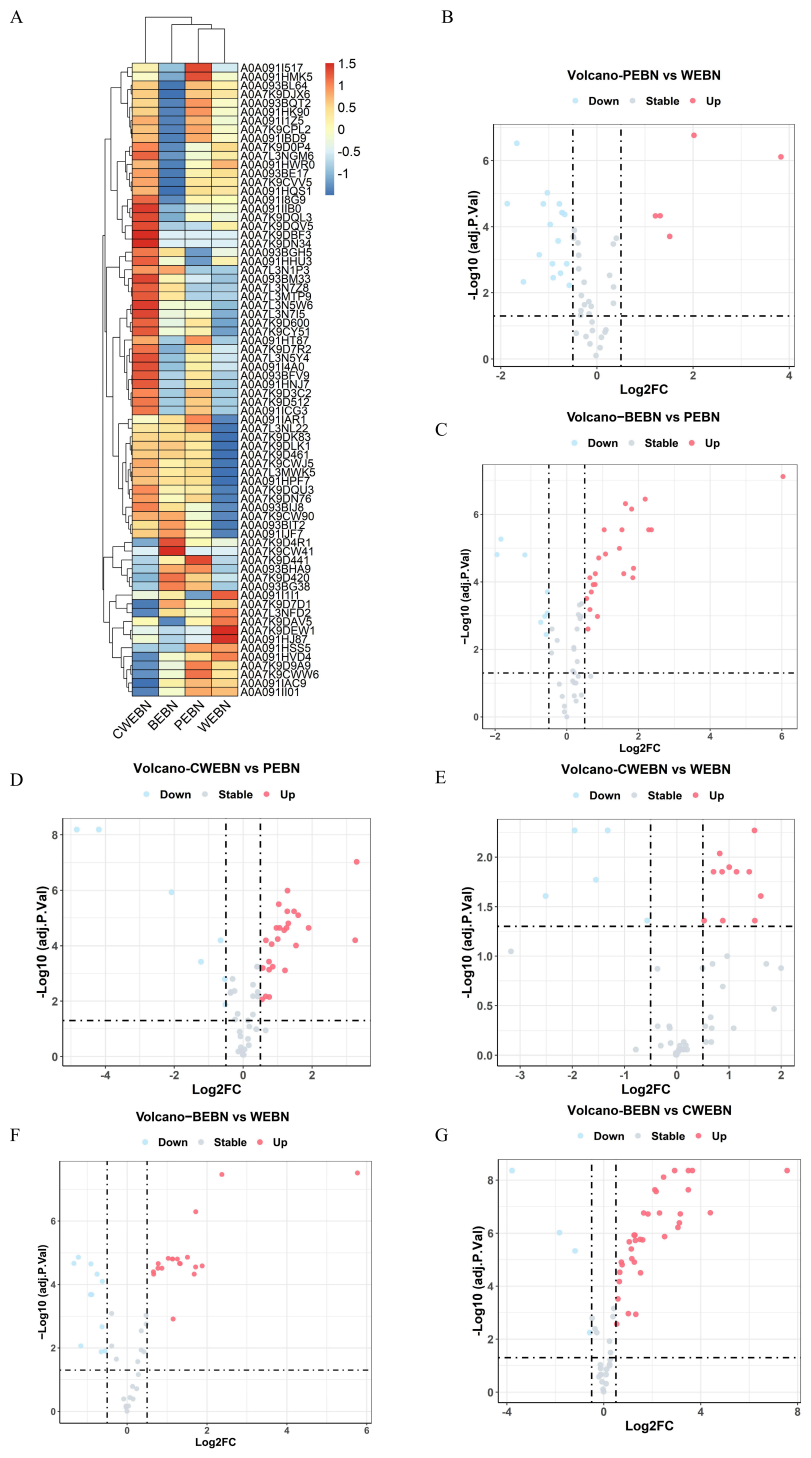
Proteomic analysis of EBN proteins. (**A**) Heatmap of differentially abundant proteins in WEBN, CWEBN, BEBN, and PEBN. Volcano plots of differentially expressed proteins in (**B**) PEBN vs. WEBN, (**C**) BEBN vs. PEBN, (**D**) CWEBN vs. PEBN, (**E**) CWEBN vs. WEBN, (**F**) BEBN vs. WEBN, and (**G**) BEBN vs. CWEBN.

The heatmap in Figure 6 shows 73 differential protein-related features identified among different colored EBNs. These features represent the proteins detected across different colored EBN samples and were used for hierarchical clustering analysis. The protein groups are different in different colored EBNs. As shown in the clustering results in Figure 6A, two main clusters of these different proteins are visible in the heatmap. Among them, the samples of BEBN, PEBN, and WEBN exhibit similar patterns, whereas those of CWEBN are distinct. In this study, proteins with *p*-values < 0.05 and |Log2 Fold Change| > 1.00 were selected as significantly different proteins. The results showed that compared with PEBN, WEBN exhibited 4 upregulated and 17 downregulated proteins; BEBN showed 23 upregulated and 8 downregulated proteins; CWEBN showed 25 upregulated and 7 downregulated proteins. Compared with WEBN, CWEBN showed 11 upregulated and 5 downregulated proteins, while BEBN showed 11 upregulated and 5 downregulated proteins. Compared with BEBN, CWEBN showed 34 upregulated and 4 downregulated proteins.

A total of 59 peptide sequences showed significant (*p* < 0.05) abundance changes between two or more colored EBNs. After removing redundant peptide sequences corresponding to the same protein, a total of 51 unique differentially abundant proteins were identified, including enzymes, catalytic proteins, binding proteins, etc. These results indicate that the 73 protein-related features identified from the proteomic dataset were further refined based on statistical significance and peptide-level abundance variation. Among these features, 59 peptides exhibited significant abundance differences among different colored EBN samples. After mapping redundant peptide sequences to their corresponding proteins, 51 unique differentially abundant proteins were identified, representing the non-redundant protein-level changes associated with EBN color variation.

These 51 unique differentially abundant proteins included proteins with enzymatic activity, catalytic functions, and binding-related functions. Together, these results demonstrate the stepwise refinement of the proteomic dataset, from differential protein-related feature screening (73 features), to significant peptide-level variation identification (59 peptide sequences), and finally to non-redundant protein-level identification (51 unique differentially abundant proteins).

**Figure 7:**
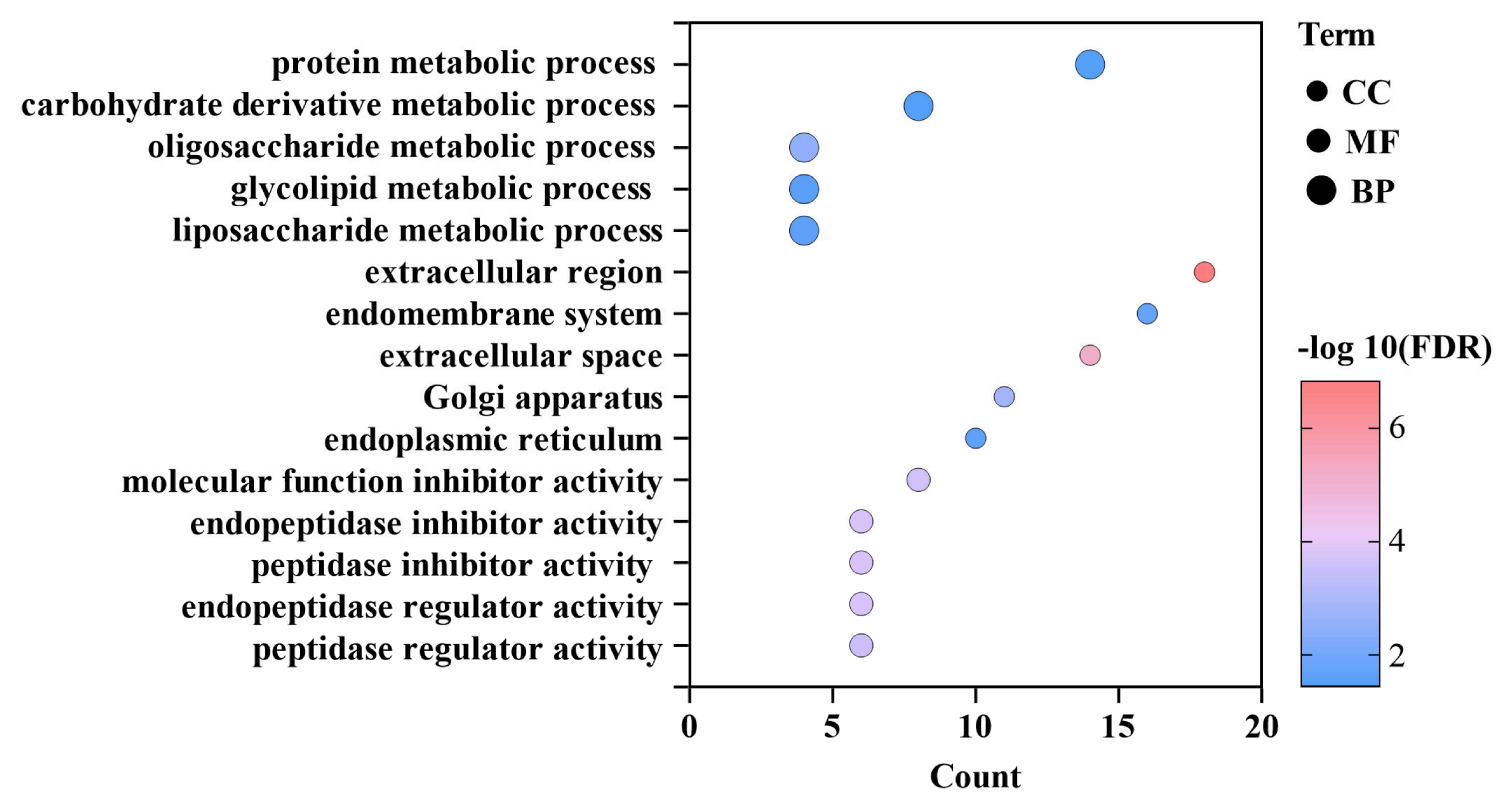
Functional analysis of EBN differentially expressed proteins.

There were significant differences in the protein content of EBNs with different colors through pairwise comparisons. To understand the potential biological functions of the different proteins in EBNs, we conducted Gene Ontology (GO) analysis. GO analysis was performed to provide preliminary functional insights into the differentially expressed proteins. Due to the limited availability of species-specific functional annotation resources for Aerodramus swiftlets, GO enrichment analysis was performed based on homologous functional annotations from the closely related avian model species Gallus gallus. GO analysis was performed based on the 51 unique differentially abundant proteins. The three major categories of GO annotation include MF, CC, and BP. The enriched BP terms included protein metabolic process, carbohydrate derivative metabolic process, oligosaccharide metabolic process, glycolipid metabolic process, and liposaccharide metabolic process. The CC annotations suggested that the differentially expressed proteins were mainly associated with extracellular region, endomembrane system, extracellular space, Golgi apparatus, and endoplasmic reticulum. The main molecular functions of MF included molecular function inhibitor activity, endopeptidase inhibitor activity, peptidase inhibitor activity, endopeptidase regulator activity, and peptidase regulator activity (Figure 7).

## 4. Discussion

### 4.1. Relationship Between Protein Characteristics and EBN Color

The objective color measurement results confirmed significant differences in color characteristics among WEBN, CWEBN, BEBN, and PEBN, supporting the validity of the four-category classification. Therefore, the classification of EBN samples in this study was not solely based on commercial naming but was supported by measurable color variations. These objectively distinguishable color phenotypes provide a basis for further investigating the relationship between protein characteristics and EBN color variation.

The results showed that the protein concentration of BEBN was higher than that of the other three EBNs, and the protein extraction rate was also higher. This might be related to the drying methods employed post-harvesting [19]. The smaller particle size of BEBN allows it to bind to the Bradford reagent dye more easily, which facilitates protein solubility. Therefore, the soluble protein content was higher in BEBN.

In terms of molecular weight distribution, the proteins in EBN primarily concentrated in the macromolecular fraction (100–170 kDa), suggesting the presence of sialic acid. In EBNs, sialic acid binds to 106 and 128 kDa proteins to form sialoglycoproteins. However, a group of common proteins with a range of 35–70 kDa was present, suggesting that proteins within this molecular weight range may represent conserved components among different colored EBN samples. Chua et al. confirmed that EBN contains a group of glycoproteins that are not influenced by the color, origin, and processing method of EBN [20].

Notably, BEBN had fewer high-molecular-weight proteins compared to the other three colors, but more proteins in the 10–35 kDa range. These differences in molecular weight distribution suggest variations in protein composition among different colored EBN samples. However, the specific factors responsible for these differences remain unclear. Previous studies have suggested that factors such as swiftlet species, environmental conditions, and processing procedures may influence EBN composition [17], but further studies are required to determine their contribution to protein variation and color formation.

These results imply that differences in protein composition may be associated with color variation among EBN samples, providing potential insights into the molecular basis underlying different EBN color phenotypes.

### 4.2. Potential Roles of Nitrite and Phenolics in EBN Coloration

The swiftlet (genus Aerodramus) usually builds its nests in limestone caves, where there is an abundance of bat feces and other organic matter. In these environments, the action of microorganisms and the associated nitrification process convert the ammonia in the organic matter into nitrate and nitrite. Meanwhile nitrites and nitrates are widely employed in the food industry as food additives and curing agents, serving to enhance quality and appearance [21]. This is due to their efficacy in extending shelf life and inhibiting the growth of several aerobic or anaerobic microorganisms. However, since nitrates can be converted into N-nitrosamines, which increases the risk of cancer and methemoglobinemia [22], it is necessary to strictly limit the acceptable daily intake of nitrate. In this study, nitrite levels in all samples were within the limits set by the government, but there were differences in nitrite content among the four types of EBNs. Reports indicate that the red coloration of EBN correlates with elevated nitrite and nitrate levels [23]. Therefore, further research is needed to explore whether there is a relationship between this difference and their color.

Regarding phenolic compounds, EBN are mainly composed of proteins and carbohydrates, different from the abundant phenolic substances in grains, which affect the color of the grain skins [24]. The overall polyphenol content of the EBN is relatively low, and its impact on coloration is likely to be minimal.

### 4.3. Mineral Composition and Color Variation

The Dietary Reference Intake (DRI) for Na in adults is 1500 mg per day, for K it is 4700 mg per day, for Mg it is 320–420 mg per day, and for Ca it is 1000 mg per day [25]. This study indicated that 10 g of EBN provides 20% of the daily dietary reference intake for Na, 0.43% for K, 42.86% to 56.25% for Mg, 0.8% for Ca, and other trace mineral elements, so EBN is regarded as a favorable source of minerals. At the same time, they are important elements for the human body. Ca helps maintain bone and tooth health, Mg helps relax muscles, and Na helps maintain the body’s water balance [26]. As minerals from limestone caves permeate into the nests, natural cave-harvested EBN exhibited relatively higher mineral content [27]. In this study, the relatively elevated levels of Na, K, and Mg in CWEBN may be associated with its cave-derived origin. The PCA results showed that the mineral composition of CWEBN and BEBN was significantly different, while there was no obvious difference between PEBN and WEBN. CWEBN exhibited relatively higher Na, K, and Mg contents, whereas BEBN showed relatively higher Fe and Ni contents.

### 4.4. Proteomic Mechanisms Underlying EBN Color Formation

The proteomic analysis revealed that the highly abundant peptide sequences identified in BEBN contained several amino acids with potential metal-chelating abilities. Basic amino acids such as arginine (R), as well as acidic amino acids including aspartic acid (D), glutamate (E), lysine (K), and histidine (H), have been reported to participate in metal ion coordination [28]. This observation was consistent with the ICP-MS results showing relatively higher Fe content in BEBN. However, the present findings only indicate a possible association between peptide composition, metal ion distribution, and EBN color variation, rather than providing direct evidence of metal–peptide complex formation or its contribution to coloration. Therefore, metal ion chelation by peptide components may represent a potential factor influencing EBN color formation, but this hypothesis requires further validation using targeted molecular interaction and spectroscopic analyses.

Among the differentially expressed proteins, A0A7K9D9A9 (Chitinase) showed significant changes. Chitin is a natural high-molecular-weight polymer widely present in the cell walls of insects and fungi [29]. Chitinase is widely present in nature and can catalyze the hydrolysis of chitin to generate N-acetylglucosamine, which is mainly responsible for the decomposition of chitin. Chitinase can be produced by different organisms and plays various physiological roles in these organisms. The presence of chitinase in bird saliva may be related to the physiological functions and structural characteristics of chitin in insects. Fiorilla et al. proved that the natural response of birds enables them to produce chitin hydrolase, which ultimately plays a role in improving the digestibility of insects [30]. In fact, chitinase is involved in the digestion process of Southeast Asian swiftlets, especially when decomposing target organisms containing chitin such as arthropods, which helps improve the digestion and absorption of nutrients in the food by the birds. In addition, acidic mammalian chitinase (AMC) (A0A093BFV9, A0A091IAC9) showed relatively significant changes, and they both belong to a glycoprotein and are the main proteins in EBN [13]. Relevant studies have suggested that Fe may interact with AMC under specific conditions, which has been proposed to be associated with red color formation in EBN [31]. However, whether similar interactions occur in different colored EBN samples remains unclear. In this study, BEBN showed relatively high Fe content but did not exhibit a red coloration phenotype. This observation suggests that Fe concentration alone may not be sufficient to explain EBN color differences, and other factors, including peptide composition, protein structure, and possible metal-binding characteristics, may also contribute to color variation. Chitinases in EBNs of all colors bear the basic physiological function of digesting insect chitin, but differences in their isoforms, abundance, or post-translational modifications may lead to different color outcomes.

A0A093BM33, A0A093BE17 and A0A7K9D3C2 derived from the deleted in malignant brain tumors protein 1 (DMBT1) were found to show differential changes in four groups of EBNs. DMBT1 belongs to the scavenger receptor rich in cysteine (SRCR) superfamily, which is considered an inhibitory gene for many cancers, and plays a role in the mucosal defense system, cellular immune defense, and epithelial cell differentiation [32]. DMBT1 is a secreted glycoprotein mainly located in epithelial cells and is recognized as a pattern recognition receptor, which binds to endogenous proteins such as IgA, surfactant proteins, and MUC5B, exhibiting the function of innate mucosal immunity [33]. In birds, the presence of DMBT1 may be similar to its function in mucosal immunity and epithelial cell differentiation. Although specific studies are limited, it can be speculated that its presence in bird saliva provides mucosal immune protection and regulates inflammatory responses. Therefore, the swiftlets that produce nests of different colors may participate in the immune response by binding to pathogens under the local inflammatory responses or immune dysregulation, resulting in significant differences in the abundance of DMBT1 in their saliva. This is related to factors such as individual birds, the environment, and other factors. This remodeling of the proteome caused by the change of immune status may indirectly affect the protein network related to color formation and become a trigger for color change.

A0A093BIT2 showed significant changes in the CWEBN, WEBN, and BEBN groups. This sequence was derived from the lysyl oxidase homolog (LOXL). It has been reported that LOXL is also present in the crude protein of extracted EBN [34]. Lysyl oxidase homolog 3 (LOXL3) mediates the deacetylation of signal transducer and activator of transcription signal transducer and activator of transcription 3(STAT3), activates cytokines or growth factors such as interleukin-6 (IL-6), and regulates the differentiation of Th17 cells, enabling them to regulate immunity [35]. LOXL3 can also stabilize fibrils and elastin elasticity. After supplementing EBN to ovariectomized rats, the skin of the rats improved significantly [36], which supports the view that intake of EBN has an effect on skin improvement and anti-aging.

A0A7K9D600 derived from the 45 kDa calcium-binding protein exhibited significant changes in four different colored EBNs. Wong et al. identified the EBN protein by shotgun method and discovered the existence of the 45 kDa calcium-binding protein [37], which was verified in this study. It has been shown that the expression of calcium-binding proteins can alleviate endoplasmic reticulum stress. It mainly participates in regulating the calcium-dependent activities in the endoplasmic reticulum cavity or posterior region [38], which may play a key role in cellular calcium homeostasis, covering aspects from calcium homeostasis to calcium signaling pathways. Besides calcium-binding proteins, 78 kDa glucose-regulated protein (GRP78) can also alleviate endoplasmic reticulum stress. In this study, A0A091HVD4 was derived from this protein. GRP78 is a member of the heat shock protein 70 family and is located on the endoplasmic reticulum membrane. It is the main calcium-binding protein. In normal cells, GRP78 can prevent the transport of misfolded proteins or protein subunits. Under endoplasmic reticulum stress conditions, when unfolded proteins accumulate in the endoplasmic reticulum cavity, GRP78 is activated and therefore alleviates endoplasmic reticulum stress by regulating cell activities and increasing endoplasmic reticulum folding ability [39].

Taken together, the proteomic differences among EBN color categories may reflect variations in the physiological status, environmental responses, and structural characteristics of swiftlets. Although several differentially expressed proteins and peptide sequences showed potential relationships with metal-binding processes and color variation, their direct roles in determining EBN coloration remain to be elucidated. The integration of proteomic, elemental, and colorimetric analyses provides new insights into the potential factors associated with EBN color diversity; however, further studies are required to confirm the specific molecular mechanisms underlying color formation.

### 4.5. Functional Implications of Differentially Expressed Proteins

The GO analysis results showed that the EBN proteins were distributed in extracellular region, endomembrane system, extracellular space, Golgi apparatus, and endoplasmic reticulum. This result was consistent with the research results of Wang et al., proving that the EBN proteins are secreted proteins [40]. The main molecular functions of MF include molecular function inhibitor activity, endopeptidase inhibitor activity, peptidase inhibitor activity, endopeptidase regulator activity, and peptidase regulator activity. The sialic acid in the EBN exists in the form of glycoproteins and is linked in a combined form with mucopolysaccharides, glycoproteins, and glycolipids of low-molecular-weight chains [41]. The significant enrichment of protease and endopeptidase inhibitor activities among the EBN proteins suggests a protective mechanism that limits unintended proteolytic cleavage. This inhibition may play a key role in stabilizing the intricate sialoglycoprotein network and preventing the enzymatic degradation of protein-bound sialic acid components within the nesting matrices. However, whether these functions have an impact on the color of the EBN still needs further research.

## 5. Conclusions

In summary, this study initially explored the differences in key indicators such as nitrite, inorganic metal element content, and protein composition among four different colored EBNs. The objective color measurements further confirmed that the four EBN categories exhibited distinguishable color characteristics, providing quantitative support for the classification of WEBN, CWEBN, BEBN, and PEBN. The results showed that differences in elemental composition, peptide characteristics, and protein profiles were associated with color variation among EBN samples. Although metal ion–peptide interactions may represent one possible factor contributing to EBN color differences, the underlying molecular mechanisms require further validation. The variation in EBN composition and color characteristics may be influenced by multiple factors, including biological differences among swiftlets, environmental conditions, and dietary sources; however, their specific contributions remain to be elucidated.

The results of this study are conducive to further standardizing the production and processing procedures of the EBN industry, providing scientific insights into the factors associated with EBN color variation, and promoting the healthy and sustainable development of the EBN industry.

## Figures and Tables

**Table 1:** Molecular weight distribution of protein in EBN.

Molecular Weight	Percentage%			
	PEBN	WEBN	CWEBN	BEBN
10~35 kDa	16.07	17.59	20.36	28.47
35~55 kDa	15.58	10.29	15.88	18.70
55~100 kDa	6.10	9.80	10.06	16.42
100~170 kDa	62.25	62.32	53.71	36.41

## Data Availability

The original contributions presented in this study are included in the article and its Supplementary Materials. The raw proteomics data generated in this study are being deposited in the ProteomeXchange Consortium via the PRIDE database under the accession number PXD081645. Further inquiries regarding the data can be directed to the corresponding authors.

## Supplementary Materials

The following supporting information can be downloaded at: https://www.mdpi.com/article/10.3390/foods15152712/s1, Figure S1: SDS-PAGE analysis of protein profiles extracted from the four types of edible bird’s nest (EBN), including PEBN, WEBN, CWEBN, and BEBN.

## References

[1] Chan K.-L., Zhang K.Y., Chan D.J.Y., Goh A.J.Y., Tan T.X.D., Tan K.W.K. (2013). Surveillance of nitrite level in cubilose: Evaluation of removal method and proposed origin of contamination. Food Control.

[2] Dong Y.-W., Chen J., Wu Y., Chen Y., Jin L. (2020). A comprehensive review of edible bird’s nest. Food Res. Int..

[3] Yap T.H., Bakar A.S., Lee S.J., Sabran S.R. (2021). A Systematic Review of Edible Swiftlet’s Nest (ESN): Nutritional bioactive compounds, health benefits as functional food, and recent development as bioactive ESN glycopeptide hydrolysate. Trends Food Sci. Technol..

[4] Cheong W.Y., Phang C.W., Lee S.J., Bakar A.S. (2022). Insights on the molecular mechanism of neuroprotection exerted by edible bird’s nest and its bioactive constituents. Food Sci. Hum. Wellness.

[5] Lim A.J.W., Chye L.S., Bakar A.S., Latip J., Kuwahara M., Lee S.J. (2021). Review of sialic acid’s biochemistry, sources, extraction and functions with special reference to edible bird’s nest. Food Chem..

[6] Chok K.C.C., Ng M.G., Ng K.Y., Koh R.Y., Tiong Y.L., Chye S.M. (2021). Edible Bird’s Nest: Recent Updates and Industry Insights Based on Laboratory Findings. Front. Pharmacol..

[7] Massimo M.F. (2005). Characterization of the edible bird’s nest the “Caviar of the East”. Food Res. Int..

[8] Brudzynski K. (2023). Unexpected Value of Honey Color for Prediction of a Non-Enzymatic H2O2 Production and Honey Antibacterial Activity: A Perspective. Metabolites.

[9] Zhang G., Xue W., Dai J., Xu Q., Wang Y., Yuan H., Yang K., Qi Y., Zeng X., Nyima T. (2019). Quantitative proteomics analysis reveals proteins and pathways associated with anthocyanin accumulation in barley. Food Chem..

[10] Pan S., Aebersold R., Clifton N.J. (2006). Quantitative proteomics by stable isotope labeling and mass spectrometry. Methods in Molecular Biology.

[11] Noordin N.N.M., Ibrahim R.M., Bakar M.Z.A., Mahmud R., Rahman N.A.A. (2021). Characterization and Extraction Influence Protein Profiling of Edible Bird’s Nest. Foods.

[12] Ma X., Zhang J., Liang J., Ma X., Xing R., Han J., Guo L., Chen Y. (2019). Authentication of Edible Bird’s Nest (EBN) and its adulterants by integration of shotgun proteomics and scheduled multiple reaction monitoring (MRM) based on tandem mass spectrometry. Food Res. Int..

[13] Liu X., Lai X., Zhang S., Huang X., Lan Q., Li Y., Li B., Chen W., Zhang Q., Hong D. (2012). Proteomic profile of edible bird’s nest proteins. J. Agric. Food Chem..

[14] Bradford M.M. (1976). A rapid and sensitive method for the quantitation of microgram quantities of protein utilizing the principle of protein-dye binding. Anal. Biochem..

[15] Ainsworth E.A., Gillespie K.M. (2007). Estimation of total phenolic content and other oxidation substrates in plant tissues using Folin–Ciocalteu reagent. Nat. Protoc..

[16] (2016). National Food Safety Standard—Determination of Nitrite and Nitrate in Foods.

[17] Guo L., Wang Y., Li M., Wang B., Gu Y., Chen Y. (2017). Determination of edible bird’s nests by FTIR and SDS-PAGE coupled with multivariate analysis. Food Control.

[18] Saengkrajang W., Matan N., Matan N. (2013). Nutritional composition of the farmed edible bird’s nest (*Collocalia fuciphaga*) in Thailand. J. Food Compos. Anal..

[19] Gan J., Chye L.S., Nor N.A.M., Bakar A.S., Lee S.J. (2020). Evaluation of physicochemical properties, amino acid profile and bioactivities of edible Bird’s nest hydrolysate as affected by drying methods. Lwt-Food Sci. Technol..

[20] Chan Y.G., Chua S.H., Chou B.C., Lau S.F.Y., Liu L.P. (2014). Identification of edible Bird’s nest with amino acid and monosaccharide analysis. J. Agric. Food Chem..

[21] Aron-Wisnewsky D., Goldberg O., Reifen R., Zelicha Hayon S., Schwartz B., Tirosh O. (2021). Whole body metabolism is improved by hemin added to high fat diet while counteracted by nitrite: A mouse model of processed meat consumption. Food Funct..

[22] Zhang Y., Yang Z., Zhang Y., Farooq S., Li Y., Zhang H. (2023). Novel Ag-coated nanofibers prepared by electrospraying as a SERS platform for ultrasensitive and selective detection of nitrite in food. Food Chem..

[23] Wong C.-F., Chan K.-L., Zhang M., Yao P., Lin H.Q., Dong T.T.X., Li G., Lai X.P., Tsim K.W.K. (2017). Characterization of edible bird’s nest by peptide fingerprinting with principal component analysis. Food Qual. Saf..

[24] Yang C., Zhu X., Liu W., Huang J., Xie Z., Yang F., Shang Q., Yang F., Wei Y. (2024). Quantitative analysis of the phenolic compounds and antioxidant activities of six quinoa seed grains with different colors. Lwt-Food Sci. Technol..

[25] Barroso M.F., Silva A., Ramos S., Oliva-Teles M.T., Delerue-Matos C., Sales M.G.F., Oliveira M.B.P.P. (2009). Flavoured versus natural waters: Macromineral (Ca, Mg, K, Na) and micromineral (Fe, Cu, Zn) contents. Food Chem..

[26] Ruan E., Sharp P. (2023). Minerals and trace elements. Human Nutrition: A Very Short Introduction.

[27] Quek M.C., Chok N.L., Yeo Y.A., Lai C.L., Tan S.W. (2018). Characterization of edible bird’s nest of different production, species and geographical origins using nutritional composition, physicochemical properties and antioxidant activities. Food Res. Int..

[28] Chen D., Wang Z.-B., Wang L.-X., Zhang P., Yang C.-H., Bao L. (2023). Structure, catalysis, chitin transport, and selective inhibition of chitin synthase. Nat. Commun..

[29] Rulíšek L., Havlas Z. (2000). Theoretical Studies of Metal Ion Selectivity. 1. DFT Calculations of Interaction Energies of Amino Acid Side Chains with Selected Transition Metal Ions (Co^2+^, Ni^2+^, Cu^2+^, Zn^2+^, Cd^2+^, and Hg^2+^). J. Am. Chem. Soc..

[30] Fiorilla E., Gariglio M., Gai F., Zambotto V., Bongiorno V., Cappone E.E., Biasato I., Bergagna S., Madrid J., Martinez-Miró S. (2024). Dehydrated and live black soldier fly larvae as environmental enrichment in indigenous slow-growing chickens: Performance, gut health, and chitinolytic enzyme activity. Anim. Int. J. Anim. Biosci..

[31] Khan S.N., Ali I., Ahmed F., Shah M.R., Shaheen F. (2023). Synthesis of conjugated silver nanoparticles of 18β-glycyrrhetinic acid peptide conjugate (GAP) as a colorimetric probe for barium ions. New J. Chem..

[32] Zhao Y., Tao Q., Wu J., Liu H. (2019). DMBT1 has a protective effect on allergic rhinitis. Biomed. Pharmacother. Biomed. Pharmacother..

[33] Ligtenberg A.J.M., Karlsson N.G., Veerman E.C.I. (2010). Deleted in malignant brain tumors-1 protein (DMBT1): A pattern recognition receptor with multiple binding sites. Int. J. Mol. Sci..

[34] Wang X., Hu D., Liao F., Chen S., Meng Y., Dai J., Dong T.T.X., Lao Z., Yu L., Liang Y. (2023). Comparative proteomic analysis of edible bird’s nest from different origins. Sci. Rep..

[35] Ma L., Huang C., Wang X.-J., Xin D.E., Wang L.-S., Zou Q.C., Zhang Y.-S., Tan M.-D., Wang Y.-M., Zhao T.C. (2017). Lysyl Oxidase 3 Is a Dual-Specificity Enzyme Involved in STAT3 Deacetylation and Deacetylimination Modulation. Mol. Cell.

[36] Lee C.H., Hamdan N., Nyakuma B.B., Wong S.L., Wong K.Y., Jamaluddin H., Lee T.H. (2024). Functional and biological activities of Edible Bird’s Nest (EBN) protein by proteomic and bioinformatic analyses. J. Food Meas. Charact..

[37] Wong Z.C.F., Chan K.-L., Wang L., Li H., Yao P., Tan T.T.-X., Tsim K.W.K. (2017). A comprehensive proteomics study on edible bird’s nest using new monoclonal antibody approach and application in quality control. J. Food Compos. Anal..

[38] He S.-W., Liang Y.-L., Zhang Y., Liu X., Gong S., Ye M.-L., Huang S.-Y., Tan X.-R., Zhou S.-Q., Zhao Y. (2023). LINC00173 facilitates tumor progression by stimulating RAB1B-mediated PA2G4 and SDF4 secretion in nasopharyngeal carcinoma. Mol. Oncol..

[39] Yuan M., Lin X., Wang D., Dai J. (2023). Proteins: Neglected active ingredients in edible bird’s nest. Chin. Herb. Med..

[40] Zhang L., Ten Hagen K.G. (2019). O-Linked glycosylation in Drosophila melanogaster. Curr. Opin. Struct. Biol..

[41] Chong P.K., Mun S.L., Chang L.S., Babji A.S., Lim S.J. (2022). Fractionation of edible bird’s nest glycoprotein hydrolysates: Characterisation and antioxidative activities of the fractions. Food Sci. Hum. Wellness.

